# Supplementary material for: Analysis of the genetically tractable crustacean Parhyale hawaiensis reveals the organisation of a sensory system for low-resolution vision
Source: BMC Biol. 2019 Aug 15;17:67. doi: 10.1186/s12915-019-0676-y (PMC6694581; doi:10.1186/s12915-019-0676-y)
Supplement: Supplementary file 1 — Opsin sequence accession numbers and spectral information. (PDF 65 kb) [file 12915_2019_676_MOESM1_ESM.pdf]

Additional file 1

Opsin sequence accession numbers and available spectral information

(taken from Porter et al. 2007)

Species	**Acession #**	**λmax**
Anolis carolinensis pineal opsin	AH007737	
Apis mellifera	AF004169	353
Apis mellifera	U26026	529
Apis mellifera	AF004168	439
Archaeomysis grebnitzkii	DQ852573	496
Bicyclus anynana	AF484249	560
Bombus terrestris	AY485301	529
Bos taurus rhodopsin	AH001149	
Calliphora erythrocephala Rh1	M58334	490
Cambarellus shufeldtii	AF003544	526
Cambarus ludovicianus	AF003543	529
Camponotus abdominalis	AF042788	360
Camponotus abdominalis	U32502	510
Cataglyphis bombycinus	U32501	510
Cataglyphis bombycinus	AF042787	360
Daphnia magna blue	KZS21495.1	
Daphnia magna LWS	KZS05019.1	
Daphnia magna UV	KZS12137.1	
Drosophila melanogaster Rh1	AH001026	478
Drosophila melanogaster Rh2	M12896	420
Drosophila melanogaster Rh3	M17718	345
Drosophila melanogaster Rh4	AH001040	375
Drosophila melanogaster Rh5	U67905	437
Drosophila melanogaster Rh6	Z86118	508
Enteroctopus dofleini	X07797	475
Euphausia superba	DQ852576	487
Galleria mellonella	AF385330	510
Gallus gallus pineal opsin	U15762	
Heliconius erato	AF126750	570
Heliconius sara	AF126753	550
Hemigrapsus sanguineus	D50583	480
Holmesimysis costata	DQ852581	512
Homarus gammarus	DQ852587	515
Homo sapiens GPR52	NM_005684	
Homo sapiens melatonin receptor	NM_005958	
Hyalella azteca	XP_018018325	
Junonia coenia	AF385332	510
Limulus Polyphemus (ocelli)	L03782	530
Limulus polyphemus (lateral eye)	L03781	520
Loligo forbesi	X56788	494
Loligo pealii	AY450853	493
Loligo subulata	Z49108	499
Manduca sexta	L78080	520
Manduca sexta	AD001674	450
Manduca sexta	L78081	357
Mysis diluviana	DQ852591	501
Neogonodactylus oerstedii Rh1	DQ646869	489
Neogonodactylus oerstedii Rh2	DQ646870	528
Neogonodactylus oerstedii Rh3	DQ646871	522
Neomysis americana	DQ852592	520
Orconectes virilis	AF003545	530
Osmia rufa	AY572828	553
Papilio xuthus Rh1	AB007423	520

Papilio xuthus Rh2	AB007424	520
Papilio xuthus Rh3	AB007425	575
Papilio xuthus Rh4	AB028217	460
Papilio xuthus Rh5	AB028218	
Pieris rapae	AB177984	540
Procambarus clarkii	S53494	533
Procambarus milleri	AF003546	522
Schistocerca gregaria	X80072	430
Schistocerca gregaria	X80071	520
Sepia officinalis	AF000947	492
Sphodromantis sp.	X71665	515
Spodoptera exigua	AF385331	515
Todarodes pacificus	X70498	480
Vanessa cardui	AF385333	530
