# Supplementary material for: Analysis of the genetically tractable crustacean Parhyale hawaiensis reveals the organisation of a sensory system for low-resolution vision
Source: BMC Biol. 2019 Aug 15;17:67. doi: 10.1186/s12915-019-0676-y (PMC6694581; doi:10.1186/s12915-019-0676-y)
Supplement: Supplementary file 2 — Trimmed alignment of opsin sequences. (DOCX 46 kb) [file 12915_2019_676_MOESM2_ESM.docx]

**Additional file 2**

**Trimmed alignment of opsin sequences**

Parhyale_Ops2 MSHSH----SPYAVPGGQAPGGPSFGYPPGVSLVDIVPPHIKDLIHPHWS

Parhyale_Ops1 MAS---WNNPASMLPE--GMVST-NPFG-NFTVVDAAPKDLLPLIDPHWY

Hyalella_azteca MSHSH----YASASRGALGANDFVFGYAPGVSVVDIVPPHMKDLIHPHWS

SW_Procambarus_clarkii MALLDGTNDTNLIRPALFGEGVAAGGRYEMRMLGWNTPSEYMDYVHPYWK

LW-like_Daphnia_magna MANLT----GDAVVAMAQKQAFDPWALPDSFTLYAYAPEDIRSFLHPHWH

UV-like_Daphnia_magna MLLPNG---NESTGPIAQ------SWKYESRMNGWNTPADYKSYVHPHWL

BLUE-like_Daphnia_magna MALNST--------------ASFDAFRSDRRMMGWNTPEDYMSYVHPYWK

494_Loligo_forbesi MGRDI-----------------------PDNETWWYNP---YMDIHPHWK

475_Enteroctopus_dofleini MVESTT----------------------LVNQTWWYNP---TVDIHPHWA

490_Calliphora_erythrocephalaR MERYS---IGPSFAAL------------TNGSVTDKVTPDMAHLVHPYWN

520_Limulus_polyphemus MANQL-------------SYSSLGWPYQPNASVVDTMPKEMLYMIHEHWY

530_L_polyphemus MANQL-------------SYSSLGWPYQPNASVVDTMPKEMLYMIHEHWY

420_D_melanogasterRh2 MERSHTAHSGPRFQAQSS----------GNGSVLDNVLPDMAHLVNPYWS

345_D_melanogasterRh3 MESGNSGNVSTALRPEA-------RLSAETRLLGWNVPPEELRHIPEHWL

533_Procambarus_clarkii MSS---WSNQPAMDDY--GLPSS-NPYG-NFTVVDMAPKDILHMIHPHWY

515_Sphodromanti_ssp MS----LINEPSYSAY--SWGGQ-GGYG-NQTVVDKVLPEMLHLIDPHWY

480_Todarodes_pacificus MGRDL-----------------------RDNETWWYNP---SIVVHPHWR

Gallus_gallus_pinealOpsin MSSNS-----------------------QAPPNGTPGPFD-----GPQWP

499_Loligo_subulata ------------------------------NETWWYNP---YMDIHSHWK

480_Hemigrapsus_sanguineus MANVT----GPQMAFY--GSGAATFGYPEGMTVADFVPDRVKHMVLDHWY

529_Apis_mellifera M-I---AVSGPSYEAF--SYGGQ-ARFN-NQTVVDKVPPDMLHLIDANWY

510_Cataglyphis_bombycinus MS----IASGPSHAAY--TWTAQGGGFG-NQTVVDKVPPEMLHLVDAHWY

510_Camponotus_abdominalis MS----IASGPSHAAY--TWASQGGGFG-NQTVVDKVPPEMLHMVDAHWY

520_Schistocerca_gregaria MASAS-LISEPSFSAY---WGGS-GGFA-NQTVVDKVPPEMLYLVDPHWY

430_S_gregaria MVNTT--DFYPVPAAMAY------ESSVGLPLLGWNVPTEHLDLVHPHWR

437_D_melanogasterRh5 MHI------NGPSGPQAY--VNDSLGDGSVFPMGHGYPAEYQHMVHAHWR

508_Drosophila_melanogasterRh6 MAS---LH-PPSFAYM---------RDGRNLSLAESVPAEIMHMVDPYWY

492_Sepia_officinalis MGRDI-----------------------PDNETWWYNP---TMEVHPHWK

529_Cambarus_ludovicianus -----------------------------------------LHMIHLHWY

526_Cambarellus_shufeldtii -----------------------------------------LHMIHLHWY

530_Orconectes_virilis -----------------------------------------LHMIHLHWY

522_Procambarus_milleri -----------------------------------------LHMIHLHWY

360_C_bombycinus MYT------NRSVHWEA-----RILPAGPPRLLGWNVPAEELVHIPEHWL

360_C_abdominalis MY-------NGSFHWEA-----RILPAGPPRLLGWNVPAEELVHIPEHXL

439_A_mellifera MLLHNTGKALAFIAEEGY------VPSMREKFLGWNVPPEYSDLVHPHWR

353_A_mellifera MS-------NDSIHWEA-----RYLPAGPPRLLGWNVPAEELIHIPEHWL

520_Papilio_xuthusRh1 MAMDS-LD--PGAASAP-AWAGKIEAYGSNHTVIDQVLPEMLHLIDPHWY

520_P_xuthusRh2 MAIAN-LE--PGMGASE-AWGGQAAAFGSNQTVVDKVTPDMMHLIDPHWY

575_P_xuthusRh3 MALNY-LN--TGAAKMD-TWNGQMSAYGANQTVVDKVLPEMLHLIDPHWY

450_M_sexta MATNF-TQELYEIGPMAY-PLKMISKDVAEHMLGWNIPEEHQDLVHDHWR

520_Manduca_sexta ------MDPGPGLAALQ-AWAAKSPAYGANQTVVDKVPPDMMHMIDPHWY

357_M_sexta MNNQS-ENYYHGAQFEAL------KSAGAIEMLGDGLTGDDLAAIPEHWL

460_P_xuthusRh4 MAANY----SDDIGPMAY-PMKLVSSEMVEHMMGWNIPEEHQAMVHAHWR

P_xuthus_Rh5 MDNHT-ENNYNYGAYFAP------YRLEGVELLGAGLTGEDLAAIPEHWL

570_Heliconius_erato -------------------------------------------------H

550_Heliconius_sara -------------------------------------------------H

510_Galleria_mellonella -------------------------------------------------Y

515_Spodoptera_exigua -------------------------------------------------Y

510_Junonia_coenia -------------------------------------------------Y

560_Bicyclus_anynana --------------------------------------------------

493_Loligo_pealii MGRDI-----------------------PDNETWWYNP---YMEINSHWK

529_Bombus_terrestris -------------------------------------------------Y

553_Osmia_rufa -------------------------------------------------Y

540_Pieris_rapae MAITN-LDPAPGVAAMQ-SFGIHAEAFGSNQTVIDKVLPEMMHLIDPHWY

489_Neogonodactylus_oerstediiR MSY---WNSNKIVEEY--SLPST-NPYG-NFTVVDTVPENMLHMIHSHWY

528_N_oerstediiRh2 MSY---YDDSASAAYT--------NPYG-NHTVVDTVPADMLHLIDSHWY

522_N_oerstediiRh3 MSY---WNSNKAMEEY--SLPST-NPYG-NFTVVDTVPENMLHMVHSHWY

496_Archaeomysis_grebnitzkii --------------------------------------------------

487_Euphausia_superba --------------------------------------------------

512_Holmesimysis_costata --------------------------------------------------

515_Homarus_gammarus --------------------------------------------------

501_Mysis_diluviana --------------------------------------------------

520_Neomysis_americana --------------------------------------------------

530_Vanessa_cardui MAITS-LD--PGAAALQ-AWGGQMAAFGSNETVVDKVLPDMLHLVDPHWY

Homo_sapiens_GPR52 MNE-------------------------------------------SRWT

Homo_sapiens_MTNR1A MQGN------------------------GSALPNASQPVLRGDGARPSW-

Anolis_carolinensis_pinealOpsi M------------------------------LNGTPGPFE-----GPQWP

478_D_melanogasterRh1 MESFA-AQLGPHFAPL------------SNGSVVDKVTPDMAHLISPYWN

375_D_melanogasterRh4 MEPLC-NASEPPLRPEA-----RSSGNGDLQFLGWNVPPDQIQYIPEHWL

Bostaurus_rhodopsin MNGTEN----------------------SNKTGVVRSPFE-----APQY-

Parhyale_Ops2 NFPPVNPMWHYLLGVIYIILGSLAIFGNGVVIYLFLKVKRLRMPSNLLVV

Parhyale_Ops1 QYPPMNPLWYGLLGFTIFILGILSIVGNAVVIWVFMNTKSLRSPANLLVV

Hyalella_azteca SFPPPNPMWHYLLGIIYIVMGVLATFGNGVVIYLYMKIKKLRTPSNLLVL

SW_Procambarus_clarkii TFQAPNPFLHYMLAVLYIMFMFAALVGNGVVIWVFTSAKNLRTPSNMFII

LW-like_Daphnia_magna TYKALHPAWYYFLGLMYLVIGTCAVAGNAVVLKIFSRFPALRSPANLLVM

UV-like_Daphnia_magna SYEEPNPMLHHLLGVLYIFFMIASCLGNGIVIYIFSTTKELKTPSNILIL

BLUE-like_Daphnia_magna TFEAPNPFLHYTLGFFYIIFMFCALMGNGVVIWIFTSCKSLRTPSNMLVV

494_Loligo_forbesi QFDQVPAAVYYSLGIFIAICGIIGCVGNGVVIYLFTKTKSLQTPANMFII

475_Enteroctopus_dofleini KFDPIPDAVYYSVGIFIGVVGIIGILGNGVVIYLFSKTKSLQTPANMFII

490_Calliphora_erythrocephalaR QFPAMEPKWAKFLAAYMVLIATISWCGNGVVIYIFSTTKSLRTPANLLVI

520_Limulus_polyphemus AFPPMNPLWYSILGVAMIILGIICVLGNGMVIYLMMTTKSLRTPTNLLVV

530_L_polyphemus AFPPMNPLWYSILGVAMIILGIICVLGNGMVIYLMMTTKSLRTPTNLLVV

420_D_melanogasterRh2 RFAPMDPMMSKILGLFTLAIMIISCCGNGVVVYIFGGTKSLRTPANLLVL

345_D_melanogasterRh3 TYPEPPESMNYLLGTLYIFFTLMSMLGNGLVIWVFSAAKSLRTPSNILVI

533_Procambarus_clarkii QYPPMNPMMYPLLLIFMLFTGILCLAGNFVTIWVFMNTKSLRTPANLLVV

515_Sphodromanti_ssp QFPPMNPLWHGLLGFVIGCLGFVSVVGNGMVIYIFSTTKGLRTPSNLLVV

480_Todarodes_pacificus EFDQVPDAVYYSLGIFIGICGIIGCGGNGIVIYLFTKTKSLQTPANMFII

Gallus_gallus_pinealOpsin --YQAPQSTYVGVAVLMGTVVACASVVNGLVIVVSICYKKLRSPLNYILV

499_Loligo_subulata QFDQVPAAVYYSLGIFIAICGIIGCAGNGIVIYLFTKTKSLQTPANMFII

480_Hemigrapsus_sanguineus NYPPVNPMWHYLLGVVYLFLGVISIAGNGLVIYLYMKSQALKTPANMLIV

529_Apis_mellifera QYPPLNPMWHGILGFVIGMLGFVSAMGNGMVVYIFLSTKSLRTPSNLFVI

510_Cataglyphis_bombycinus QFPPMNPLWHAILGFVIGILGMISVIGNGMVIYIFTTTKSLRTPSNLLVI

510_Camponotus_abdominalis QFPPMNPLWHALLGFVIGVLGVISVIGNGMVIYIFTTTKSLRTPSNLLVV

520_Schistocerca_gregaria QFPPMNPLWHGLLGFVIGVLGVISVIGNGMVIYIFSTTKSLRTPSNLLVV

430_S_gregaria SFQVPNKYWHFGLAFVYFMLMCMSSLGNGIVLWIYATTKSIRTPSNMFIV

437_D_melanogasterRh5 GFREAPIYYHAGFYIAFIVLMLSSIFGNGLVIWIFSTSKSLRTPSNLLIL

508_Drosophila_melanogasterRh6 QWPPLEPMWFGIIGFVIAILGTMSLAGNFIVMYIFTSSKGLRTPSNMFVV

492_Sepia_officinalis QFNQVPDAVYYSLGIFIGICGIIGCTGNGIVIYLFTKTKSLQTPANMFII

529_Cambarus_ludovicianus QYPPMNPMMYPLLLVFMLITGILCLAGNFVTIWVFMNTKSLRTPANLLVV

526_Cambarellus_shufeldtii QYPPMNPMMYPLLLIFMFITGIPCLAGNFVTIWVFMTTKSLRSPANLLVV

530_Orconectes_virilis QYPPMNPMMYPLLLIFMLFTGILCLAGNFVTIWVFMNTKSLRTPANLLVV

522_Procambarus_milleri QYPPMNPMMYPLLLIFMLFTGILCLAGNFVTIWVFMNTKSLRTPANLLVV

360_C_bombycinus VYPEPNPSLHYLLAILYTLFTFVALLGNGLVIWIFISAKSLRTPSNMFVV

360_C_abdominalis VYPEPNPSLHYLLAIVYILFTFVALFGNGLVIWIFCSAKSLRTPSNLFVV

439_A_mellifera AFPAPGKHFHIGLAIIYSMLLIMSLVGNCCVIWIFSTSKSLRTPSNMFIV

353_A_mellifera VYPEPNPSLHYLLALLYILFTFLALLGNGLVIWIFCAAKSLRTPSNMFVV

520_Papilio_xuthusRh1 QFPPMNPLWHGLLGFVIAVLGFISLSGNGMVIYIFTTTKTLKTPSNLLVL

520_P_xuthusRh2 QFPPMNPMWHGLLGFTIGVLGFISITGNGMVVYIFTSTKSLKTPSNLLVV

575_P_xuthusRh3 QFPPMNPLWYGLLGFTITCLAITSITGNAMVIYIFTTTKNLKTPSNLLVV

450_M_sexta NFPAVSKYWHYVLALIYTMLMVTSLTGNGIVIWIFSTSKSLRSASNMFVI

520_Manduca_sexta QFPPMNPLWHALLGFTIGVLGFVSISGNGMVIYIFMSTKSLKTPSNLLVV

357_M_sexta SYPAPPASAHTALALLYIFFTFAALVGNGMVIFIFSTTKSLRTSSNFLVL

460_P_xuthusRh4 SFPAVSKYYHFILALIYTMLMVTSLVGNGIVIWIFSTSKSLRSASSMFVI

P_xuthus_Rh5 SYPAPPASAHTMLALVYVFFTAAALIGNGLVIFIFSASKSLRTPSNLLVV

570_Heliconius_erato QFPPMNPLWHGLLGFVIGVLGFISVTGNGMVVYIFTTTKTLKTPSNILVV

550_Heliconius_sara QFPPMNPLWHGLLGFVIGVLGFISVTGNGMVVYIFTTTKSLKTPSNILVV

510_Galleria_mellonella QFPPMNPLWHGLLGFTIGVLGFISITGNGMVIYIFSSTKTLKTPSNLLVV

515_Spodoptera_exigua QFPPMNPLWHGLLGFTIGVLGFISITGNGMVIYIFMSTKSLKTPSNLLVV

510_Junonia_coenia QFPPMNPMWHGLLGFFMAVIGFISIAGNGMVVYIFTTTKSLKTPSNILVV

560_Bicyclus_anynana --------------------------------------------------

493_Loligo_pealii QFDQVPAAVYYSLGIFIGICGIIGCVGNGIVIYLFTKTKSLQTPANMFII

529_Bombus_terrestris QFPPLNPMWHGILGFVIGLLGFISVSGNGMVVYIFLSTKSLRTPSNMFVI

553_Osmia_rufa QYPPLNPLWHAILGLVIGILGFISVSGNGMVVYIFLSTKSLRTPSNLFVI

540_Pieris_rapae QFPPLNPLWHALLGFTISVLAFISITGNGMVVYIFTTTKSLKTPSNLLVV

489_Neogonodactylus_oerstediiR QFPPLNPMWYGILAFVVTVVGLCSICGNFVVIWVFMNTKALRSPANTLVV

528_N_oerstediiRh2 GFPPLNDMWYGILAFFISVTGILAVGGNFVVIWVFKCTKALRSPLNYYVV

522_N_oerstediiRh3 QFPPLNPMWYGILAFVVTVVGLCSICGNFVVIWVIMNTKALRSPANTLVV

496_Archaeomysis_grebnitzkii ----MNPLWYGLLGFVIFCLGILSVCGNAVVIWVFMNTKSLRSPANLLVV

487_Euphausia_superba ----MNPLWYGLLGFVIFCLGCLSVFGNSVVIWVFTSTKTLRSPANMLVV

512_Holmesimysis_costata ----MNPLWYGLLGFWMTVMGTLSVAGNFVVIWVFMNTKSLRTPANLLVV

515_Homarus_gammarus ----MNPLWYGLLALWMFVMGTLSVCGNSIVIWVFMNTKALRTPANLLVV

501_Mysis_diluviana ----MKSRWYIILGLIISVLAILSVIGNLTVIVVFINTRSLRSPSNLLIV

520_Neomysis_americana ----MNPLWYSLVGFWMVIMGVLSVVGNFVVLWVFMTTKSLRTPANLLVV

530_Vanessa_cardui QFPPMNPLWHGLLGFVIGILGFISITGNGMVIYIFTTTKSLKTPSNILVV

Homo_sapiens_GPR52 EWRILNGFGHYSVTVVIVLLTFLIIAGNLTVIFVFHCAPLLHYTTSYFIQ

Homo_sapiens_MTNR1A --------LASALACVLIFTIVVDILGNLLVILSVYRNKKLRNAGNIFVV

Anolis_carolinensis_pinealOpsi --FLAPRGTYTSVAVLMGLVVVLATVVNGLVIVVSVRYKRLRSPLNYILV

478_D_melanogasterRh1 QFPAMDPIWAKILTAYMIMIGMISWCGNGVVIYIFATTKSLRTPANLLVI

375_D_melanogasterRh4 TQLEPPASMHYMLGVFYIFLFCASTVGNGMVIWIFSTSKSLRTPSNMFVL

Bostaurus_rhodopsin --YLAEPWQFSMLAAYMFLLIMLGFPINFLTLYVTVQHKKLRTPLNYILL

Parhyale_Ops2 NLAIMDMLMLLSQFPFFVWNCFNGVWMFSPFMCELYACFGSVSGLGSLWT

Parhyale_Ops1 NLAVSDFLMMLTMFPPMVVSCYYHTWAFSPFFCELYGFFGSIFGCISIWT

Hyalella_azteca NLAIMDMLMLLSQFPFFVYNCFHGVWAFSPFMCELYACFGAISGLGSLWT

SW_Procambarus_clarkii NLAILDFIMML-KTPVFIVNSFNEGPIWGKLGCDTFALMGSYSGVGGAVT

LW-like_Daphnia_magna NLAVSDFLLMLALFPECVYNFFLGPWRFGEMGCQIHAFLGACFGYNQIFT

UV-like_Daphnia_magna NLAICDFIMMI-KTPVFIVNSFNEGPVFGRLGCSIYGLLGAYVGPCSAVT

BLUE-like_Daphnia_magna NLAILDFIMMM-KTPIFIMNSYNEGPIWGKLGCDVFALLGSYNGIGSAMN

494_Loligo_forbesi NLAFSDFTFSLNGFPLMTISCFMKYWVFGNAACKVYGLIGGIFGLMSIMT

475_Enteroctopus_dofleini NLAMSDLSFSANGFPLKTISAFMKKWIFGKVACQLYGLLGGIFGFMSINT

490_Calliphora_erythrocephalaR NLAISDFGIMITNTPMMGINLFYETWVLGPLMCDIYGGLGSAFGCSSILS

520_Limulus_polyphemus NLAFSDFCMMAFMMPTMTSNCFAETWILGPFMCEVYGMAGSLFGCASIWS

530_L_polyphemus NLAFSDFCMMAFMMPTMASNCFAETWILGPFMCEVYGMAGSLFGCASIWS

420_D_melanogasterRh2 NLAFSDFCMMASQSPVMIINFYYETWVLGPLWCDIYAGCGSLFGCVSIWS

345_D_melanogasterRh3 NLAFCDFMMMV-KTPIFIYNSFHQGYALGHLGCQIFGIIGSYTGIAAGAT

533_Procambarus_clarkii NLAMSDFLMMFTMFPPMMVTCYYHTWTLGPTFCQVYAFLGNLCGCASIWT

515_Sphodromanti_ssp NLAFSDFLMMLSMSPPMVINCYYETWVLGPFMCELYALLGSLFGCGSIWT

480_Todarodes_pacificus NLAFSDFTFSLNGFPLMTISCFLKKWIFGFAACKVYGFIGGIFGFMSIMT

Gallus_gallus_pinealOpsin NLAVADLLVTLCGSSVSLSNNINGFFVFGRRMCELEGFMVSLTGIVGLWS

499_Loligo_subulata NLAFSDFTFSLNGFPMMTISCFLKHWVFGQAACKVYGLIGGIFGLTSIMT

480_Hemigrapsus_sanguineus NLALSDLIMLTTNFPPFCYNCFSGRWMFSGTYCEIYAALGAITGVCSIWT

529_Apis_mellifera NLAISNFLMMFCMSPPMVINCYYETWVLGPLFCQIYAMLGSLFGCGSIWT

510_Cataglyphis_bombycinus NLAISDFLMMLSMSPAMVINCYYETWVLGPLVCELYGLTGSLFGCGSIWT

510_Camponotus_abdominalis NLAISDFLMMLCMSPAMVINCYYETWVLGPLFCELYGLAGSLFGCASIWT

520_Schistocerca_gregaria NLAFSDFLMMFTMSAPMGINCYYETWVLGPFMCELYALFGSLFGCGSIWT

430_S_gregaria NLALFDVLMLL-EMPMLVVSSFYQRPVGWELGCDIYAALGSVAGIGSAIN

437_D_melanogasterRh5 NLAIFDLFMCT-NMPHYLINATVGYIVGGDLGCDIYALNGGISGMGASIT

508_Drosophila_melanogasterRh6 NLAFSDFMMMFTMFPPVVLNGFYGTWIMGPFLCELYGMFGSLFGCVSIWS

492_Sepia_officinalis NLAFSDFTFSLNGFPLMTISCFIKKWVFGMAACKVYGFIGGIFGLMSIMT

529_Cambarus_ludovicianus NLAMSDFLMMFTMFPPMMITCYYHTWTLGATFCEVYAFLGNLCGCASIWT

526_Cambarellus_shufeldtii NLAMSDFLMMFTMFPPMMITCYYHTWTLGPTFCQVYAFLGNLFGCTSIWT

530_Orconectes_virilis NLAMSDFLMMFTMFPPMMVTCYYHTWTLGPTFCQVYAFLGNLCGCASIWT

522_Procambarus_milleri NLAMSDFLMMFTMFPPMMVTCYYHTWTLGPTFCQVYGFLGNLCGCASIWT

360_C_bombycinus NLAFCDFIMML-KAPIFIYNSFNTGFATGHLGCQIFACMGALSGIGASMT

360_C_abdominalis NLAFCDFMMML-KAPIFIYNSFHTGFATGHLGCQIFACMGSLSGIGAGMT

439_A_mellifera SLAIFDIIMAF-EMPMLVISSFMERMIGWEIGCDVYSVFGSISGMGQAMT

353_A_mellifera NLAICDFFMMI-KTPIFIYNSFNTGFALGNLGCQIFAVIGSLTGIGAAIT

520_Papilio_xuthusRh1 NLAVSDFLMMTCMAPPLVVNSYHETWVFGPLACALYAAAGSLFGTISIWT

520_P_xuthusRh2 NLAFSDFLMMLCMAPPMLINCYYETWVFGPLACELYACAGSLFGSISIWT

575_P_xuthusRh3 NLAVSDFLMMACMAPPLIINSYNETWVFGPLFCAIYACGGSLYGTVSIWT

450_M_sexta NLAVFDLMMML-EMPLLIMNSFYQRLVGYQLGCDVYAVLGSLSGIGGAIT

520_Manduca_sexta NLAFSDFLMMCAMSPAMVVNCYYETWVWGPFACELYACAGSLFGCASIWT

357_M_sexta NLAILDFIMMA-KAPIFIYNSAMRGFAVGTVGCQIFALMGAYSGIGAGMT

460_P_xuthusRh4 NLAVFDLMMMI-EMPLLIANSFYQHPIGFQLGCDVYAVLGSISGIGGAIT

P_xuthus_Rh5 QLAVLDFLMML-KAPIFIYNSIKRGFASGVIGCQIFAFMGSVSGTAAGLT

570_Heliconius_erato NLAFSDFLMMFMMAPPMVINCYNETWVFGPLACQLYACAGSLYGCVSIWT

550_Heliconius_sara NLAFSDFLMMFMMAPPMVINCYNETWVFGPLACQLYACAGSLYGCVSIWT

510_Galleria_mellonella NLAFSDFLMMCAMSPAMVVNCYYETWVFGPFACELYGCAGSLFGCTSIWT

515_Spodoptera_exigua NLAFSDFLMMCCMSPAMVVNCYNETWVWGPLACELYACAGSLFGCASIWT

510_Junonia_coenia NLAFSDFLMMTVMSPPMVVSCYYETWVFGPLACQLYACAGSLCGCASIWT

560_Bicyclus_anynana --ALFDFLMMLVXSPPMVVNCYNETWVFGPLACQLYACAGSLFGCTSIWT

493_Loligo_pealii NLAFSDFTFSLNGFPLMTISCFLKYWVFGNAACKVYGLIGGIFGLMSIMT

529_Bombus_terrestris NLAISDFLMMFCMSPPMVINCYYETWVLGPLFCQVYAMLGSLFGCGSIWT

553_Osmia_rufa NLAISDFLMMFCMSPPMIINCYFETWVFGPLFCQIYAMLGSLFGCSSIWT

540_Pieris_rapae NLAFSDFLMMAMMAPPLVVNSYNETWVFGPTACQFYACFGSLFGCVSIWT

489_Neogonodactylus_oerstediiR SLAVSDFIMMACMFPPLVLNCYWGTWIFGPLFCEVYAFIGNTVGCASIGN

528_N_oerstediiRh2 NLAISDFTLMACMCPPVVINSYYRTWIFGPTFCYVYAAIGSLTGCASIFT

522_N_oerstediiRh3 SLAVSDYIMMTCMFPPLVLNCYWGTWIFGPLFCEVYAFIGNTVGCASIGN

496_Archaeomysis_grebnitzkii NLAFSDFLMMLNMFPPMVHSCYHGTWMLGAFFCEFYGFTGSLFGCISIWT

487_Euphausia_superba NLALSDFLMMANMSPPTVHSCYHGTWMLGPTYCEYYALVGSLSGCISIWT

512_Holmesimysis_costata NLAISDFFMMLTMTPPLLANAYWGTWILGAFFCEVYAFLGSFFGCVSIWS

515_Homarus_gammarus NLAISDFLMMFCMCPPLLINCYYQTWVWGAFACEVYGCIGSTVGTCSIFC

501_Mysis_diluviana NLAFSDFFMMCNMCPAMLLACIYKTWLLGPTYCAWYAFSGSLFGCLSIWT

520_Neomysis_americana NLALSDFLMMFTMFPPMVISCYWQTWTLGAFFCEVYAFLGSLFGCVSIWS

530_Vanessa_cardui NLAFSDFLMMCVMSPPMVVNCYTETWVFGPLACQLYACAGSLFGCASIWT

Homo_sapiens_GPR52 TMAYADLFVGVCLVPTLSLL-HYSTGVHESLTCQVFGYIISVLKSVSMAC

Homo_sapiens_MTNR1A SLAVADLVVAIYPYPLVLMSIFNNGWNLGYLHCQVSGFLMGLSVIGSIFN

Anolis_carolinensis_pinealOpsi NLAVADLLVTSFGSTISFANNIYGFFVFGPTACEFEGFMVSLTGIVGLWS

478_D_melanogasterRh1 NLAISDFGIMITNTPMMGINLYFETWVLGPMMCDIYAGLGSAFGCSSIWS

375_D_melanogasterRh4 NLAVFDLIMCL-KAPIF--NSFHRGFALGNTWCQIFASIGSYSGIGAGMT

Bostaurus_rhodopsin NLAVADLFMVFGGFTTTLYTSLHGYFVFGPTGCNLEGFFATLGGEIALWS

Parhyale_Ops2 LVFISYDRYNVIVHGVGG-KPLTFTKAALFLLFVWSYAIAISLPPFFG-W

Parhyale_Ops1 MIFITLDRYNVIVKGVSA-EPLTSKGALLRIMFVWVVSTVWTILPFFG-W

Hyalella_azteca LVFISYDRYNVIVKGVGG-KPLSFGKAMMCLVFVWGYATAISLPPFFG-W

SW_Procambarus_clarkii NAAIAYDRYKTIAKPFEA--KISRGTALMMVVGIWAYASPWALLPLFNIW

LW-like_Daphnia_magna LTMISYDRYNVIVKGFSG-TPLTFNRAVTIITMSWIWALGWSICPLVG-W

UV-like_Daphnia_magna NAAIAYDRYRCISDPMGK--RWSKSQASLIVLGCWVYASPVSLLPFTELV

BLUE-like_Daphnia_magna NAAIAYDRHRTIARPLDG--KLTRKQVTLMIVAIWAWATPFSILPFFGIW

494_Loligo_forbesi MTMISIDRYNVIGRPMSASKKMSHRKAFIMIIFVWIWSTIWAIGPIFG-W

475_Enteroctopus_dofleini MAMISIDRYNVIGRPMAASKKMSHRRAFLMIIFVWMWSIVWSVGPVFN-W

490_Calliphora_erythrocephalaR MCMISLDRYNVIVKGMAG-QPMTIKLAIMKIALIWFMASIWTLAPVFG-W

520_Limulus_polyphemus MVMITLDRYNVIVRGMAA-APLTHKKATLLLLFVWIWSGGWTILPFFG-W

530_L_polyphemus MVMITLDRYNVIVRGMAA-APLTHKKATLLLLFVWIWSGGWTILPFFG-W

420_D_melanogasterRh2 MCMIAFDRYNVIVKGING-TPMTIKTSIMKILFIWMMAVFWTVMPLIG-W

345_D_melanogasterRh3 NAFIAYDRFNVITRPMEG--KMTHGKAIAMIIFIYMYATPWVVACYTETW

533_Procambarus_clarkii MVFITFDRYNVIVKGVAG-EPLSTKKASLWILTIWVLSITWCIAPFFG-W

515_Sphodromanti_ssp MVMIALDRYNVIVKGLAA-KPMTNKTAMLRILGIWAMSIAWTVFPLFG-W

480_Todarodes_pacificus MAMISIDRYNVIGRPMAASKKMSHRRAFIMIIFVWLWSVLWAIGPIFG-W

Gallus_gallus_pinealOpsin LAILALERYVVVCRPLGD-FQFQRRHAVSGCAFTWGWALLWSTPPLLG-W

499_Loligo_subulata MTMISIDRYNVIRRPMSASKKMSHRKAFIMIVFVWIWSTIWAIGPIFG-W

480_Hemigrapsus_sanguineus LCMISFDRYNIICNGFNG-PKLTQGKATFMCGLAWVISVGWSLPPFFG-W

529_Apis_mellifera MTMIAFDRYNVIVKGLSG-KPLSINGALIRIIAIWLFSLGWTIAPMFG-W

510_Cataglyphis_bombycinus MTMIAFDRYNVIVKGLSA-KPMTINGALLRILGIWFFSLGWTIAPMFG-W

510_Camponotus_abdominalis MTMIAFDRYNVIVKGLSA-KPMTINGALIRILTIWFFTLAWTIAPMFG-W

520_Schistocerca_gregaria MTMIALDRYNVIVKGLSA-KPMTNKTAMLRILFIWAFSVAWTIMPLFG-W

430_S_gregaria NAAIAFDRYRTISCPIDG--RLTQGQVLALIAGTWVWTLPFTLMPLLRIW

437_D_melanogasterRh5 NAFIAFDRYKTISNPIDG--RLSYGQIVLLILFTWLWATPFSVLPLFQIW

508_Drosophila_melanogasterRh6 MTLIAYDRYCVIVKGMAR-KPLTATAAVLRLMVVWTICGAWALMPLFG-W

492_Sepia_officinalis MSMISIDRYNVIGRPMAASKKMSHRRAFLMIIFVWMWSTLWSIGPIFG-W

529_Cambarus_ludovicianus MVFITFDRYNVIVKGVAG-EPLSTKKASLWILTVWVLSFTWCVAPFFG-W

526_Cambarellus_shufeldtii MVFITFDRYNVIVKGVAG-EPLSNKKAALWILSAWVLSFSWCSAPFFG-W

530_Orconectes_virilis MVFITFDRYNVIVKGVAG-EPLSNKKAAMWILSVWVLSTAWCMAPFFG-W

522_Procambarus_milleri MVFITFDRYNVIVKGVAG-EPLSTKKASLWILIVWVLSLAWCMAPFFG-W

360_C_bombycinus NAAIAYDRYSTIARPLDG--KLSRGQVILLIALIWTYTIPWALMPLMHVW

360_C_abdominalis NAAIAYDRYSTIARPLDG--KLSRGQVLLLIMLIWTYTIPWALMPLMQVW

439_A_mellifera NAAIAFDRYRTISCPIDG--RLNSKQAAVIIAFTWFWVTPFTVLPLLKVW

353_A_mellifera NAAIAYDRYSTIARPLDG--KLSRGQVILFIVLIWTYTIPWALMPVMGVW

520_Papilio_xuthusRh1 MTMIAFDRYNVIVKGIAA-KPMTNNGALLRILAIWVSSLAWTVAPMFG-W

520_P_xuthusRh2 MTMIAFDRYNVIVKGIAA-KPMTINGALLRILGIWLFSLAWTIAPMLG-W

575_P_xuthusRh3 MTAIAFDRYNVIVKGIAA-KPMSINGALLRILAIWLSSLAWTVAPIFG-W

450_M_sexta NAVIAFDRYKTISSPLDG--RINTVQAGLLIAFTWFWALPFTILPAFRIW

520_Manduca_sexta MTMIAFDRYNVIVKGIAA-KPMTSNGALLRILGIWVFSLAWTLLPFFG-W

357_M_sexta NACIAYDRHSTITRPLDG--RLSEGKVLLMVAFVWIYSTPWALLPLLKIW

460_P_xuthusRh4 NAVIAFDRYKTISCPLDG--RINKVQASLLIAFTWFWSMPFTILPALKVW

P_xuthus_Rh5 NACIAYDRHSTITRPLDG--RLSRGKVLLMMVCVWLYTAPWAILPQLQIW

570_Heliconius_erato MTMIAFDRYNVIVKGIAA-KPMTINGALLRVFGIWAFSLAWTIAPLFG-W

550_Heliconius_sara MTMIAFDRYNVIVKGIAA-KPMTINGALLRVFGIWAFSLAWTIAPLFG-W

510_Galleria_mellonella MTMIAFDRYNVIVKGIAA-KPMTNNGALLRILGIWAFSLAWTLAPFFG-W

515_Spodoptera_exigua MTMIAFDRYNVIVKGIAA-KPMTNNGALLRILGIWAFSLAWTLAPFFG-W

510_Junonia_coenia MTMIAFDRYNVIVKGIAA-KPMTINGALLRVFGIWMFALAWTLAPMFG-W

560_Bicyclus_anynana MTMIAFDRYNVIVKGIAG-KPLTINGALLRVFAIWLFSLAWTIAPLFG-W

493_Loligo_pealii MTMISIDRYNVIGRPMSASKKMSHRKAFIMIIFVWIWSTTWAIGPIFG-W

529_Bombus_terrestris MTMIAFDRYNVIVKGLSG-KPLTINGALLRILGIWLFSLIWTIAPMFG-W

553_Osmia_rufa MTAIAFDRYNVIVKGLSG-RPLTINGALLRLLGIWLFSLIWTVAPVLG-W

540_Pieris_rapae MTAIAFDRYNVIVKGIAA-KPMTINSALLRILGVWLFSLAWTLAPIFG-W

489_Neogonodactylus_oerstediiR MIFITFDRYNVIVKGISG-TPLSQKNTTLQVLFVWICSIMWCVFPFFG-W

528_N_oerstediiRh2 MCLISYDRYNVIVKGIGG-KPSPPARQMLIILLVWLFKPPGTFAPFFG-W

522_N_oerstediiRh3 MIFITFDRYNVIVKGVSG-KPLSQKNATLQVLFVWICSIMWCVFPFFG-W

496_Archaeomysis_grebnitzkii MVFITMDRYNVIVKGVAA-EPLTSKGASIRILFVWTVAFAWTILPFFG-W

487_Euphausia_superba MVWITLDRYNVIVKGVAA-TPLTNKGAFARNIFSWLSALIWCVSPLYG-W

512_Holmesimysis_costata MVFITADRYNVIVKGVSA-EPLTSGGAMMRIAGTWAFTLAWCLPPFFG-W

515_Homarus_gammarus MVFITMDRYNVIVKGVSA-TPLTTNGAMLRNLFSWVTSIGWCLPPFFG-F

501_Mysis_diluviana MVWITLERYNVIVKGVSS-KPLSVKGAITRIVLTWIFAVIWCSFPLVG-W

520_Neomysis_americana MVWITLDRYNVIVKGVSG-EPLTNSGAMTRIAGTWVTAFAWCLPPFFG-W

530_Vanessa_cardui MTMIAFDRYNVIVKGIAA-KPLTINGAMLRVLGIWVFSLAWTVAPLFG-W

Homo_sapiens_GPR52 LACISVDRYLAITKPLSYNQLVTPCRLRICIILIWIYSCLIFLPSFFG-W

Homo_sapiens_MTNR1A ITGIAINRYCYICHSLKYDKLYSSKNSLCYVLLIWLLTLA-AVLPNLR-A

Anolis_carolinensis_pinealOpsi LAILAFERYLVICKPVGD-FRFQQRHAVIGCAFTWLWSLLWTLPPLFG-W

478_D_melanogasterRh1 MCMISLDRYQVIVKGMAG-RPMTIPLALGKM-------------------

375_D_melanogasterRh4 NAAIGYDRYNVITKPMNR--NMTFTKAVIMNIIIWLYCTPWVVLPLTQFW

Bostaurus_rhodopsin LVVLAIERYVVVCKPMSN-FRFGENHAIMGVAFTWVMALACAAPPLVG-W

Parhyale_Ops2 GRYIPEGILDSCSFDYLSRPWSIRSHGVFLFVCCYCVPLCTILYSYIFIV

Parhyale_Ops1 NRYVPEGNMTACGTDYLSESAFSKSYLYIYGSWVYMLPLIIIIVAYSQIV

Hyalella_azteca GRYIPEGILDSCSFDYLSRDWSIRSHGVFLFFFCYCVPLSTILYSYVYIV

SW_Procambarus_clarkii GRFVPEGFLTTCTFDYMSEDASTRAFVGSIFVFAYIVPGSLVFYFYGQIF

LW-like_Daphnia_magna GAYAMDGIMGTCSYDYVSQNMNNKSHILAATFANYILPIIIIAGCYYFIV

UV-like_Daphnia_magna NRFVPEGYLTSCTFDYMADNLETKIFVFLLWIWCWIMPLGVIIFSYGKIT

BLUE-like_Daphnia_magna GRFVPEGFLTTCTFDYMTEDSSTRFFVGTIFFYSYIIPLALLIFYYSKIV

494_Loligo_forbesi GAYTLEGVLCNCSFDYITRDTTTRSNILCMYIFAFMCPIVVIFFCYFNIV

475_Enteroctopus_dofleini GAYVPEGILTSCSFDYLSTDPSTRSFILCMYFCGFMLPIIIIAFCYFNIV

490_Calliphora_erythrocephalaR SRYVPEGNLTSCGIDYLERDWNPRSYLIFYSIFVYYLPLFLICYSYWFII

520_Limulus_polyphemus SRYVPEGNLTSCTVDYLTKDWSSASYVVIYGLAVYFLPLITMIYCYFFIV

530_L_polyphemus SRYVPEGNLTSCTVDYLTKDWSSASYVIIYGLAVYFLPLITMIYCYFFIV

420_D_melanogasterRh2 SAYVPEGNLTACSIDYMTRMWNPRSYLITYSLFVYYTPLFLICYSYWFII

345_D_melanogasterRh3 GRFVPEGYLTSCTFDYLTDNFDTRLFVACIFFFSFVCPTTMITYYYSQIV

533_Procambarus_clarkii NRYVPEGNLTGCGTDYLSEDILSRSYLYDYSTWVYYLPL-LPIYCYVSII

515_Sphodromanti_ssp NRYVPEGNMTACGTDYLNKEWVSRSYILVYSVFVYFLPLATIIYSYWFIV

480_Todarodes_pacificus GAYTLEGVLCNCSFDYISRDSTTRSNILCMFILGFFGPILIIFFCYFNIV

Gallus_gallus_pinealOpsin SSYVPEGLRTSCGPNWYTGGSNNNSYILSLFVTCFVLPLSLILFSYTNLL

499_Loligo_subulata GAYQLEGVLCNCSFDYITRDASTRSNIVCMYIFAFMFPIVVIFFCYFNIV

480_Hemigrapsus_sanguineus GSYTLEGILDSCSYDYFTRDMNTITYNICIFIFDFFLPASVIVFSYVFIV

529_Apis_mellifera NRYVPEGNMTACGTDYFNRGLLSASYLVCYGIWVYFVPLFLIIYSYWFII

510_Cataglyphis_bombycinus NRYVPEGNMTACGTDYLTKDLLSRSYILVYSFFCYFLPLFLIIYSYFFII

510_Camponotus_abdominalis NRYVPEGNMTACGTDYLTKDLFSRSYILIYSIFVYFTPLFLIIYSYFFII

520_Schistocerca_gregaria NRYVPEGNMTACGTDYLTKDWVSRSYILVYSFFVYLLPLGTIIYSYFFIL

430_S_gregaria SRFTAEGFLTTCSFDYLTDDEDTKVFVGCIFAWSYAFPLCLICCFYYRLI

437_D_melanogasterRh5 GRYQPEGFLTTCSFDYLTNTDENRLFVRTIFVWSYVIPMTMILVSYYKLF

508_Drosophila_melanogasterRh6 NRYVPEGNMTACGTDYFAKDWWNRSYIIVYSLWVYLTPLLTIIFSYWHIM

492_Sepia_officinalis GAYVLEGVLCNCSFDYITRDSATRSNIVCMYIFAFCFPILIIFFCYFNIV

529_Cambarus_ludovicianus NRYVPEGNLTGCGTDYLSEDILSRSYLYIYSTWVYFLPLAITIYCYVFII

526_Cambarellus_shufeldtii NRYVPEGNLTGCGTDYLSEDALSRSYLYVYSVWVYFLPLLITIYCYVFII

530_Orconectes_virilis NSYVPEGNLTGCGTDYLSEDILSRSYLYIYSTWVYFLPLTITIYCYVFII

522_Procambarus_milleri NRYVPEGNLTGCGTDYLSEDILSRSYLYIYSTWVYFLPLTITIYCYVFII

360_C_bombycinus GRFVPEGFLTSCTFDYLTDTPEIRYFVATIFTFSYCIPMSLIIYYYSQIV

360_C_abdominalis GRFVPEGFLTSCSFDYLTDSQEIRYFVPTIFTFSYCVPMLLIIYYYSQIV

439_A_mellifera GRYTTEGFLTTCSFDFLTDDEDTKVFVTCIFIWAYVIPLIFIILFYSRLL

353_A_mellifera GRFVPEGFLTSCSFDYLTDTNEIRIFVATIFTFSYCIPMILIIYYYSQIV

520_Papilio_xuthusRh1 NRYVPEGNMTACGTDYLNKDWFSRSYIVAYAIFCYFTPLALIIYSYFFII

520_P_xuthusRh2 NRYVPEGNMTACGTDYLSKSWLSRSYILVYSIFVYYTPLLLIIYSYFFIV

575_P_xuthusRh3 NRYVPEGNMTVCGTDYLSKDWLSRSYIIAYAVFCYFLPLGLIVYSYWFII

450_M_sexta GRFVPEGFLTTCSFDYFTEDQDTEVFVACIFVWSYCIPMALICYFYSQLF

520_Manduca_sexta NRYVPEGNMTACGTDYLSKSWVSRSYILIYSVFVYFLPLLLIIYSYFFIV

357_M_sexta GRYVPEGYLTSCSFDYLTNTFDTKLFVACIFTCSYVFPMSLIIYFYSGIV

460_P_xuthusRh4 GRFVPEGFLTTCSFDYFTDDQDTKVFVACIFVWSYAIPMALICYFYSQLF

P_xuthus_Rh5 GRYVPEGFLTSCTFDYLTTTFDNKLFVASMFVCVYIFPMIAILYFYSGIV

570_Heliconius_erato GRYVPEGNMTACGTDYFDQSFSNRSYILLYSIACYYAPLFLIIYSYFFIV

550_Heliconius_sara GRYVPEGNMTACGTDYFDQSFSNRSYILLYSIACYYAPLFLIIYSYFFIV

510_Galleria_mellonella NRYVPEGNMTACGTDYLNKEWLSRSYILIYSVFVYFTPLLLIIYSYFFIA

515_Spodoptera_exigua NRYVPEGNMTACGTDYLSKDWFSRSYILIYSVFVYFMPLLLIIYSYFFIV

510_Junonia_coenia GRYVPEGNMTACGTDYFDKTWFNRSYILIYSLFCYFSPLFLIIYSYFFIV

560_Bicyclus_anynana GRYVPEGNMTACGTDYFDKSWQNRSYILFYSIFCYYSPLLLICYSYFFII

493_Loligo_pealii GAYSLEGVLCNCSFDYISRDSSTRSNIVCMYLFAFMCPIIVIFFCYFNIV

529_Bombus_terrestris NRYVPEGNMTACGTDYFSKDIVSVSYILLYSIWVYFFPLFLIIWSYWFIX

553_Osmia_rufa NRYVPEGNMTACGTDYFTKDFSSISYIVMYSIWVYLLPLFLIIWSYWFII

540_Pieris_rapae SRYVPEGNMTACGTDYLSKDWASRSYIILYAIACYFLPLFLIVYSYWFIV

489_Neogonodactylus_oerstediiR NRYVPRGDMTACGTDYLTEDEFSRSYLYVYSVWVYIGPLALIIYCYFHIV

528_N_oerstediiRh2 SRYVPEGNMTACGTDYLRGSILDQTYLWSYTTWCYFMTFVFIVYCYWFIV

522_N_oerstediiRh3 NRYVPEGNMTACGTDYLTEDEFSRSYLYIYSVWVYIGPLALIIYCYFHIV

496_Archaeomysis_grebnitzkii NRYVPEGNLTACGTDYLTEDSTSHLYLYMYASWAYYTPLLYIIYAYTFIV

487_Euphausia_superba NRYVPEGNMTACGTDYLTDDWLSHSYLYAYTFWVYLFPFFIIVYCYTYIV

512_Holmesimysis_costata NRYVPEGNMLACGTDYLTETELSRSYLYVYSVWVYLFPLAYIIYSYTFIV

515_Homarus_gammarus NAYVPEGNLIACGTDYLKESVPYHVYLYLYSVWCYFLPLVIIVYCYTYIV

501_Mysis_diluviana NRYVPEGNLTACGTDYLSDDIYSQSYIYLYSVMVYFIPLGITIYCYSYIV

520_Neomysis_americana NRYVPEGNMTACGTDYLTDDKFSHSYLYIYSVWVYIFPLFLNIYLYTFII

530_Vanessa_cardui GRYVPEGNMTACGTDYLDKSWFNRSYILIYSIFCYFSPLFLIIYSYFFIV

Homo_sapiens_GPR52 GKPGYHGDIFECATSWLT-----SAYFTGFIVCLLYAPAAVVCFTYFHIF

Homo_sapiens_MTNR1A GTLQYDPRIYSCTF----AQSVSSAYTIAVVVFHFLVPMIIVIFCYLRIW

Anolis_carolinensis_pinealOpsi SSYIPEGLRTSCGPNWYTGGNDNNSYIMTLFVTCFITPLAMIIFSYANLL

478_D_melanogasterRh1 --YVPEGNLTSCGIDYLERDWNPRSYLIFYSIFVYYIPLFLICYSYWFII

375_D_melanogasterRh4 DRFVPEGYLTSCSFDYLSDNFDTRLFVGTIFFFSFVCPTLMILYYYSQIV

Bostaurus_rhodopsin SRYIPEGMQCSCGIDYYTEETNNESFVIYMFVVHFIIPLIVIFFCYGQLV

Parhyale_Ops2 KAIVSHEKAMRAQAKKMNVTNLRSGKDDGGQSAEMRVAKVACINVTLWLI

Parhyale_Ops1 SAVFAHEKQMREQAKKMGVKSLRSEEA-QKTSAECRLAKVALMTVSLWFI

Hyalella_azteca KAIISHEKAMREQAKKMNVTNLRSGKDDGGQSAEMRVAKVACINVTLWLV

SW_Procambarus_clarkii VHVRAHEQAMREQAKKMNVANLRSSHEDQEKSVEIRIAKVCMGLFFLFLI

LW-like_Daphnia_magna HAVFKHEEELRAQAKKMNVASLRSNNDQQQVSAEIRIAKVSIMNVSMWLT

UV-like_Daphnia_magna TQVMTHEARLKEQAKKMNVETLRSGANKDVRN-EIRVAKVGISLTTLFLL

BLUE-like_Daphnia_magna QSVGDHEKTLRDQAKKMNVTSLRSNRDQNEKSAEVRIAKVAIALATLFVV

494_Loligo_forbesi MSVSNHEKEMAAMAKRLNAKELRKAQ--AGANAEMKLAKISIVIVTQFLL

475_Enteroctopus_dofleini MSVSNHEKEMAAMAKRLNAKELRKAQ--AGASAEMKLAKISMVIITQFML

490_Calliphora_erythrocephalaR AAVSAHEKAMREQAKKMNVKSLRSSED-ADKSAEGKLAKVALVTISLWFM

520_Limulus_polyphemus HAVAEHEKQLREQAKKMNVASLRAADQ-QKQSAECRLAKVAMMTVGLWFM

530_L_polyphemus HAVAEHEKQLREQAKKMNVASLRAADQ-QKQSAECRLAKVAMMTVGLWFM

420_D_melanogasterRh2 AAVAAHEKAMREQAKKMNVKSLRSSED-CDKSAEGKLAKVALTTISLWFM

345_D_melanogasterRh3 GHVFSHEKALRDQAKKMNVESLRSNVDKNKETAEIRIAKAAITICFLFFC

533_Procambarus_clarkii KAVAAHEKGMRDQAKKMGIKSLRNEEA-QKTSAECRLAKIAMTTVALWFI

515_Sphodromanti_ssp QAVSAHEKQMREQAKKMNVASLRSAEN-ANTSAECKLAKVALMTISLWFF

480_Todarodes_pacificus MSVSNHEKEMAAMAKRLNAKELRKAQ--AGANAEMRLAKISIVIVSQFLL

Gallus_gallus_pinealOpsin LTLRAAAAQQKEAD--------------TTQRAEREVTRMVIVMVMAFLL

499_Loligo_subulata MSVSNHEKEMAAMAKRLNAKELRKAQ--AGASAEMKLAKISIVIVTQSLL

480_Hemigrapsus_sanguineus KAIFAHEAAMRAQAKKMNVTNLRSNEA-ETQRAEIRIAKTALVNVSLWFI

529_Apis_mellifera QAVAAHEKNMREQAKKMNVASLRSSEN-QNTSAECKLAKVALMTISLWFM

510_Cataglyphis_bombycinus QAVAAHEKNMREQAKKMNVASLRSAEN-QSTSAECKLAKVALMTISLWFM

510_Camponotus_abdominalis QAVAAHEKNMREQAKKMNVASLRSAEN-QSTSAECKLAKVALMTISLWFM

520_Schistocerca_gregaria QAVSAHEKQMREQRKKMNVASLRSAEA-SQTSAECKLAKVALMTISLWFF

430_S_gregaria GAVREHEKMLRDQAKKMNVKSLQSNADTEAQSAEIRIAKVALTIFFLFLC

437_D_melanogasterRh5 THVRVHEKMLAEQAKKMNVKSLSANANADNMSVELRIAKAALIIYMLFIL

508_Drosophila_melanogasterRh6 KAVAAHEKAMREQAKKMNVASLRNSEA-DKSKAEIKLAKVALTTISLWFF

492_Sepia_officinalis MAVSNHEKEMAAMAKRLNAKELRKAQ--AGASAEMKLAKISIVIVTQFLL

529_Cambarus_ludovicianus KAVAAHEKGMRDQAKKMGIKSLRNEEA-QKTSAECRLAKIAMTTVALWFI

526_Cambarellus_shufeldtii KAVAAHEKGMRDQAKKMGIKSLRNEEA-QKTSAECRLAKIAMTTVALWFI

530_Orconectes_virilis KAVAAHEKGMRDQAKKMGIKSLRNEEA-QKTSAECRLAKIAMTTVALWFI

522_Procambarus_milleri KAVAAHEKGMRDQAKKMGIKSLRNEEA-QKTSAECRLAKIAMTTVALWFI

360_C_bombycinus SHVVNHEKALREQAKKMNVESLRSNTNTNAQSAEIRIAKAAITICFLFVL

360_C_abdominalis GHVVSHEKALREQAKKMNVESLRSNVNTNAQSAEIRIAKAAITICFLFVL

439_A_mellifera SSIRNHEKMLREQAKKMNVKSLVSNQD-KERSAEVRIAKVAFTIFFLFLL

353_A_mellifera SHVVNHEKALREQAKKMNVDSLRSNANTSSQSAEIRIAKAAITICFLYVL

520_Papilio_xuthusRh1 QAVAAHEKAMREQAKKMNVASLRSSEA-ANTSAECKLAKVALMTISLWFM

520_P_xuthusRh2 QAVAAHEKAMREQAKKMNVASLRSSEA-ANTSAECKLAKVALMTISLWFM

575_P_xuthusRh3 QAVAAHEKAMREQAKKMNVASLRSSDA-ANTSAECKLAKVALMTISLWFM

450_M_sexta GAVRLHERMLQEQAKKMNVKSLASNKEDNSRSVEIRIAKVAFTIFFLFIC

520_Manduca_sexta QAVAAHEKAMREQAKKMNVASLRSSEA-ANTSAECKLAKVALMTISLWFM

357_M_sexta KQVFAHEAALREQAKKMNVESLRANQGGSSESAEIRIAKAALTVCFLFVA

460_P_xuthusRh4 GAVRLHERMLQEQAKKMNVKSLASNKEDASKSVEIRIAKVAFTIFFMFVC

P_xuthus_Rh5 KQVFAHEAALREQAKKMNVDSLRSNQNAAAESAEIRIAKAALTVCFLYVA

570_Heliconius_erato QAVAAHEKAMREQAKKMNVASLRSSDA-ANTSAECKLAKVALMTISLWFM

550_Heliconius_sara QAVAAHEKAMREQAKKMNVASLRSSDA-ANTSAECKLAKVALMTISLWFM

510_Galleria_mellonella QAVAAHEKAMREQAKKMNVASLRSSEA-ANTSAECKLAKVALMTISLWFM

515_Spodoptera_exigua QAVAAHEKGMREQAKKMNVASLRSSEA-ANTSAECKLAKVALMTISLWFM

510_Junonia_coenia QAVAAHEKAMREQAKKMNVASLRSSDA-ANTSAECKLAKVALMTISLWFM

560_Bicyclus_anynana QAVAAHEKAMREQAKKMNVASLRSSEN-ANTSAECKLAKVALMTISLWFM

493_Loligo_pealii MSVANHXKEMAAMAKRLNGKELRRAQ--AGASAEMKLGKISVVIVTQFLL

529_Bombus_terrestris QAVAAHEKNMREQAKKMNVASLRSSEN-QNTSAECKLAKVALMTISLWFM

553_Osmia_rufa QAVAAHEKNMREQAKKMNVASLRSSEN-QNTSAECKLAKVALMTISLWFM

540_Pieris_rapae QAVAAHERAMREQAKKMNVASLRSSEQ-ANTSAECKLAKVALMTISLWFM

489_Neogonodactylus_oerstediiR SAVATHEKQMRDQAKKMGVKSLRTEEA-KKTSAECRLAKVALTTVSLWFM

528_N_oerstediiRh2 AAVRNHEKAMREQAKKMGVKSLRGDATLQKKSADCKLAKIALINVSLWFM

522_N_oerstediiRh3 SAVATHEKQMRDQAKKMGVKSLRTEEA-KKTSAGCRLAKVALTTVSLWFM

496_Archaeomysis_grebnitzkii QAVSAHEKGMREQAKKMGVKSLRNEEA-QKTSAECRLAKVALMTVSLWFM

487_Euphausia_superba SAVFAHEKGMRDQAKKMGVKSLRNEEA-QKTSAECRLAKVALVTVSLWFI

512_Holmesimysis_costata KAVAAHEKGMREQAKKMGVKSLRSEEA-QKTSAECRLCKVALMTVTLWFM

515_Homarus_gammarus AAVSAHERQMREQAKKMGVKSLRSEES-KKTSNECRLAKVALTTVSLWFI

501_Mysis_diluviana HAVANHEKSMKEQAKKMGVKSFRNEET-QRTSAEFRLAKIALMTVSLWFI

520_Neomysis_americana KAVANHEKQMREQAKKMGVKSLRSEES-QKTSAECRLAKVALMTVSLWFM

530_Vanessa_cardui QAVAAHEKAMREQAKKMNVASLRSSDA-ANTSAECKLAKVALMTISLWFM

Homo_sapiens_GPR52 KICRQHTKEINDRRARFPSHEVDSSRE-TGHSPDRRYAMVLFRITSVFYM

Homo_sapiens_MTNR1A ILVLQVRQRVKPDRKKLKPQ-------------DFRNFVTMFVVFVLFAI

Anolis_carolinensis_pinealOpsi LTLRAVAAQQKEMA--------------TTQQAEREVTRMVVTMVMAFLV

478_D_melanogasterRh1 AAVSAHEKAMREQAKKMNVKSLRSSED-AEKSAEGKLAKVALVTITLWFM

375_D_melanogasterRh4 GHVFSHEKALREQAKKMNVESLRSNVDKSKETAEIRIAKAAITICFLFFV

Bostaurus_rhodopsin FTVKEAAAQQQESA--------------TTQKAEKEVTRMVIIMVIAFLI

Parhyale_Ops2 CWTPYAAIVLQGLFFDQSTITPLVSMLPALLAKSTACYNPMVYALSHPRF

Parhyale_Ops1 AWTPYLVINFTGMNNK-SVISPLFTIWGSLFAKANAVYNPIVYAISHPKY

Hyalella_azteca CWTPYAAIVLQGLFFDQSSITPLVSMLPALLCKTTACYNPMVYALSHPRF

SW_Procambarus_clarkii SWTPYAVVALIAAFGDRSKLTPLVSMIPALTCKFVACVDPWVYAINHPRY

LW-like_Daphnia_magna AWTPFAVICILGTWGDVSKITPLVSAIPVILAKTSCAYNPLIYAISHPKY

UV-like_Daphnia_magna SWTPYFMIAFIGCYGNRALLTPGLSMIPACTCKLAACVDPFVYAINHPKY

BLUE-like_Daphnia_magna AWTPYAFVALTAAFGNRGVLTPLMSMIPACCCKGVACINPWVYAINHPRY

494_Loligo_forbesi SWSPYAVVALLAQFGPIEWVTPYAAQLPVMFAKASAIHNPMIYSVSHPKF

475_Enteroctopus_dofleini SWSPYAIIALLAQFGPAEWVTPYAAELPVLFAKASAIHNPIVYSVSHPKF

490_Calliphora_erythrocephalaR AWTPYTIINTLGLFKY-EGLTPLNTIWGACFAKSAACYNPIVYGISHPKY

520_Limulus_polyphemus AWTPYLIISWAGVFSSGTRLTPLATIWGSVFAKANSCYNPIVYGISHPRY

530_L_polyphemus AWTPYLIIAWAGVFSSGTRLTPLATIWGSVFAKANSCYNPIVYGISHPRY

420_D_melanogasterRh2 AWTPYLVICYFGLFKI-DGLTPLTTIWGATFAKTSAVYNPIVYGISHPKY

345_D_melanogasterRh3 SWTPYGVMSLIGAFGDKTLLTPGATMIPACACKMVACIDPFVYAISHPRY

533_Procambarus_clarkii AWTPYLLINWVGMFAR-SYLSPVYTIWGYVFAKANAVYNPIVYAISHPKY

515_Sphodromanti_ssp AWTPYLVTDFSGIFEW-GKISPLATIWCSLFAKANAVYNPIVYGISHPKY

480_Todarodes_pacificus SWSPYAVVALLAQFGPLEWVTPYAAQLPVMFAKASAIHNPMIYSVSHPKF

Gallus_gallus_pinealOpsin CWLPYSTFALVVATHKGIIIQPVLASLPSYFSKTATVYNPIIYVFMNKQF

499_Loligo_subulata SWSPYAIVALLAQFGPIEWVTPYAAQLPVMFAKASAIHNPMIYSVSHPKF

480_Hemigrapsus_sanguineus CWTPYAAITIQGLLGNAEGITPLLTTLPALLAKSCSCYNPFVYAISHPKF

529_Apis_mellifera AWTPYLVINFSGIFNL-VKISPLFTIWGSLFAKANAVYNPIVYGISHPKY

510_Cataglyphis_bombycinus AWTPYLVINYAGIFET-VKINPLFTIWGSLFAKANAVYNPIVYGISHPKY

510_Camponotus_abdominalis AWTPYLVINYSGIFET-TKISPLFTIWGSLFAKANAVYNPIVYGISHPKY

520_Schistocerca_gregaria GWTPYLIINFTGIFET-MKISPLLTIWGSLFAKANAVFNPIVYGISHPKY

430_S_gregaria SWTPYAVVAMIGAFGNRAALTPLSTMIPAVTAKIVSCIDPWVYAINHPRF

437_D_melanogasterRh5 AWTPYSVVALIGCFGEQQLITPFVSMLPCLACKSVSCLDPWVYATSHPKY

508_Drosophila_melanogasterRh6 AWTPYTIINYAGIFES-MHLSPLSTICGSVFAKANAVCNPIVYGLSHPKY

492_Sepia_officinalis SWSPYAVVALLAQFGPIEWVTPYAAQLPVMFAKASAIHNPLIYSVSHPKF

529_Cambarus_ludovicianus AWTPYLLINWVGMFAR-SYLSPVYTIWGYVFAKANAVYNPIVYAIS----

526_Cambarellus_shufeldtii AWTPYLLINWVGMFAR-SYLSPVYTIWGYVFAKANAVYNPIVYAIS----

530_Orconectes_virilis AWTPYLLINWVGMFAR-SYLSPVYTIWGYVFAKANAVYNPIVYAIS----

522_Procambarus_milleri AWTPYLLINWVGMFAR-SYLSPVYTIWGYVFAKANAVYNPIVYAIS----

360_C_bombycinus SWTPYGTLAMIGAFGNKALLTPGVTMIPACTCKFVACLDPYVYAISHPRY

360_C_abdominalis SWTPYGALAMIGAFGNRALLTPGITMIPACACKFVACLDPYVYAISHPRY

439_A_mellifera AWTPYATVALIGVYGNRELLTPVSTMLPAVFAKTVSCIDPWIYAINHPRY

353_A_mellifera SWTPYGVMSMIGAFGNKALLTPGVTMIPACTCKAVACLDPYVYAISHPKY

520_Papilio_xuthusRh1 AWTPYLVINFTGIFET-ATISPLGTIWGSVFAKANAVYNPIVYGISHPKY

520_P_xuthusRh2 AWTPYLVINYTGVFET-APISPLATIWGSVFAKANAVYNPIVYGISHPKY

575_P_xuthusRh3 AWTPYLVINFAGVFET-APISPVSTIWGSVFAKANAVYNPIVYGISHPKY

450_M_sexta AWTPYAFVTMTGAFGDRTLLTPIATMIPAVCCKVVSCIDPWVYAINHPRY

520_Manduca_sexta AWTPYLVINYTGVFES-APISPLATIWGSLFAKANAVYNPIVYGISHPKY

357_M_sexta SWTPYGVMALIGAFGNQQLLTPGVTMIPAVACKAVACISPWVYAIRHPMY

460_P_xuthusRh4 GWTPYAFVTMTGAYGDRSLLTPVATMIPAVCCKIVSCIDPWVYAINHPRY

P_xuthus_Rh5 SWTPYGVMSLIGAFGDQNLLTPGVTMIPALACKGVACIDPWVYAISHPKY

570_Heliconius_erato AWTPYLVINYAGIFKT-MTISPIVTIWGSVFAKANAV-------------

550_Heliconius_sara AWTPYLVINYAGIFKT-MT-------------------------------

510_Galleria_mellonella AWTPYLAINYTGVFES-APISPLATIWGSLFAKAN---------------

515_Spodoptera_exigua AWTPYLVINYAGVFES-ATISPLATIWGSLFAKAN---------------

510_Junonia_coenia AWTPYLVINYSGIFET-ATITPLATIWGSVFAKAN---------------

560_Bicyclus_anynana AWTPYLVINYAGIFET-MTISPLVTIWGSVFAKANAVYNPFVYGIGSR--

493_Loligo_pealii SGSPYAMVALLAQFGPLEWVTRYAAQLPVMFAKASAIHNPMIYSVSHPKF

529_Bombus_terrestris AWTPYLVINWSGIFSL-VKISPLYTIWGSLFAKANAV-------------

553_Osmia_rufa AWTPYLVINFSGCFEL-VKISPLFTIWGSLFAKANAV-------------

540_Pieris_rapae AWTPYLVINFAGVFET-SPISPLSTIWGSVFAKANAVYNPIVYGISHPKY

489_Neogonodactylus_oerstediiR AWTPYLIINWAGMFYP-SVVSPLFSIWGSVFAKANAVYNPIVYAISHPKY

528_N_oerstediiRh2 AWTPYAIINIAGLTNK-EIVTPLFFIWGSVFAKANTVYNPIEYAISHPKY

522_N_oerstediiRh3 AWTPYLIINWAGMFYP-SVVSPLFSIWGSVFAKSNAVYNPIVYAISHPKY

496_Archaeomysis_grebnitzkii AWTPYMIINFTGMNDR-TKLTPLCTIWGSL--------------------

487_Euphausia_superba AWTPYCVINVTGMWDK-TKITPLFTIWGSL--------------------

512_Holmesimysis_costata AWTPYFIINWGGMFNK-PMVTPLFS-------------------------

515_Homarus_gammarus AWTPYLIINWAGMINK-PSVSPLLTI------------------------

501_Mysis_diluviana AWTPYLVINIVGMVAR-QQLNPLSTI------------------------

520_Neomysis_americana AWTPYFIINWAGMLSK-SNVTPLFSIWGSV--------------------

530_Vanessa_cardui AWTPYLVINYAGIFET-ATITPLATIWGSVFAKANAVYNPIVYGISHPKY

Homo_sapiens_GPR52 LWLPYIIYFLLES--SRVLDNPTLSFLTTWLAISNSFCNCVIYSLSNSVF

Homo_sapiens_MTNR1A CWAPLNFIGLAVASDP-ASMVPRIPEWSYYMAYFNSCLNAIIYGLLNQNF

Anolis_carolinensis_pinealOpsi CWLPYASFAMVVATNKDLLIQPALASLPSYFSKTATVYNPIIYVFMNKQF

478_D_melanogasterRh1 AWTPYLVINCMGLFKF-EGLTPLNTIWGACFAKSAACYNPIVYGISHPKY

375_D_melanogasterRh4 SWTPYGVMSLIGAFGDKSLLTQGATMIPACTCKLVACIDPFVYAISHPRY

Bostaurus_rhodopsin CWLPYAGVAFYIFTHQGSDFGPIFMTIPAFFAKTSAVYNPVIYIMMNKQF

Parhyale_Ops2 RQAMQTEVPCCCVQEPDDSTSDAKSTATEQQN---ESK-----------

Parhyale_Ops1 RAALEKKLPCLSCSGGDGASDSVSVTTTQSEQPVTEKSESA--------

Hyalella_azteca RQAMMTEIPCCCVHEPDDSTAESKSAVTEQQV---AEK-----------

SW_Procambarus_clarkii RLELQKRMPWFCIHEEKPQDTISQSTCETEKA-----------------

LW-like_Daphnia_magna RECLKQMFPWMCIVEEKKAAADNQSVISDKTEMEIVKCESA--------

UV-like_Daphnia_magna RLELMKRLPWLCVHEKDECAKEESSNASVISE---AESRT---------

BLUE-like_Daphnia_magna RMELQKKMPWFCVHEPVPSDDSSLASATTEMS-GVAKETSS--------

494_Loligo_forbesi RERIASNFPWILTDEKEIEDDKDAEAEIPAGEQGGETADAAQMKEMMAP

475_Enteroctopus_dofleini REAIQTTFPWLLTDEKECEDANDAEEEVVASERGGESRDAAQMKEMMAP

490_Calliphora_erythrocephalaR GIALKEKCPCCVFGKVDDGKASDATSQATNNE---SETKA---------

520_Limulus_polyphemus KAALYQRFPSLACGSGESGSDVKSEASATTTME--EKPKIP--------

530_L_polyphemus KAALYQRFPSLACGSGESGSDVKSEASATMTME--EKPKSP--------

420_D_melanogasterRh2 RIVLKEKCPMCVFGNTDEPKPAPASDTETTSE---ADSKA---------

345_D_melanogasterRh3 RMELQKRCPWLALNEKAPESSAVASTSTTQEP---QQTTAA--------

533_Procambarus_clarkii RAAMEKKLPCLSCKTESDDVSESASTTTSSAE---EKAESA--------

515_Sphodromanti_ssp RAALNKKFPSLACASEPDDTASQASGATTVSD---EKSASA--------

480_Todarodes_pacificus REAISQTFPWVLTDDKETEDDKDAETEIPAGESAAPSADAAQMKEMMAP

Gallus_gallus_pinealOpsin QSCLLEML---CCGYQ-PQRTGKASPGTPGPH---ADVTAAGLRNKVMP

499_Loligo_subulata REAIASNFPWILTDEKEIEDDKDAEAEIPAAEQGGESVDAAQMKEMMAP

480_Hemigrapsus_sanguineus RLAITQHLPWFCVHEKDPNDVEENQSSNTQTQ---EKS-----------

529_Apis_mellifera RAALFAKFPSLACAAESSDAVSTTSGTTTVTDN--EKSNA---------

510_Cataglyphis_bombycinus RAALFQRFPSLACSSGPAGADTLSTTTTVTEGT--EKPAA---------

510_Camponotus_abdominalis RAALFQKFPSLACTTEPTGADTMSTTTTVTEGN--EKPAA---------

520_Schistocerca_gregaria RAALEKKFPSLACASSSDDNTSVASGATTVSD---EKSEKSA-------

430_S_gregaria RAEVQKRMKWLHLGEDARSSKSDTSSTATDRT---VGNVSASA------

437_D_melanogasterRh5 RLELERRLPWLGIREKHATSGTSGGQESVASV---SGDTLALSV-----

508_Drosophila_melanogasterRh6 KQVLREKMPCLACGKDDLTSDSRTQATAEISE---SQA-----------

492_Sepia_officinalis REAIAENFPWIITDEKEVEDDKDAETEIPATEQGGESADAAQMKEMMAP

529_Cambarus_ludovicianus -------------------------------------------------

526_Cambarellus_shufeldtii -------------------------------------------------

530_Orconectes_virilis -------------------------------------------------

522_Procambarus_milleri -------------------------------------------------

360_C_bombycinus RLELQKRLPWLELQEKPI-ETQSTTTETVNTA---SS------------

360_C_abdominalis RLELQKRLPWLELQEKPVADTQSTTTEMVHTP---AS------------

439_A_mellifera RQELQKRCKWMGIHEPETTSDATSAQTEKIKTD----------------

353_A_mellifera RLELQKRLPWLELQEKPISDSTSTTTETVNTP---PASS----------

520_Papilio_xuthusRh1 RAALYQRFPSLACQPAADDNTSQVSGKTAVCE---EKPSA---------

520_P_xuthusRh2 RAALYQKFPSLACQPSAEETGSVASGATTACE---EKPSA---------

575_P_xuthusRh3 RAALYQRFPSLACQPSPDESGSVASGNTAVCE---EKAPA---------

450_M_sexta RAELQKRLPWMGVREQDPDAVSTTTSVATAGF---QPPAAEA-------

520_Manduca_sexta QAALYAKFPSLQCQSAPEDAGSVASGTTAVSE---EKPAA---------

357_M_sexta RQELQRRMPWLQIDEPDDTVSTATSNTTNSAP---PAATA---------

460_P_xuthusRh4 RAELQKRLPWLGVREQDPDTVSNSNSVTTTQS---HTPTAEA-------

P_xuthus_Rh5 RQELQKRMPWLQIDEPDDNASNTTSNTANSSA---PA------------

570_Heliconius_erato -------------------------------------------------

550_Heliconius_sara -------------------------------------------------

510_Galleria_mellonella -------------------------------------------------

515_Spodoptera_exigua -------------------------------------------------

510_Junonia_coenia -------------------------------------------------

560_Bicyclus_anynana -------------------------------------------------

493_Loligo_pealii REAIASNFPWILTDEKEIEDEKDAEAEIPACEQGGESADAAQMKEMMAP

529_Bombus_terrestris -------------------------------------------------

553_Osmia_rufa -------------------------------------------------

540_Pieris_rapae RAALYQRFPALACQPSAEETGSVASAATACTE---EKPSA---------

489_Neogonodactylus_oerstediiR RAALYKKLPCLACSTESADEGSATNSATTTTA---EKYESA--------

528_N_oerstediiRh2 KGALYQKLPWLQCAPDVPEDDSKSTASTTTTG---GEEKA---------

522_N_oerstediiRh3 RAALYKKLPCLACSTESADEGSATNSTTTATA---EKYESA--------

496_Archaeomysis_grebnitzkii -------------------------------------------------

487_Euphausia_superba -------------------------------------------------

512_Holmesimysis_costata -------------------------------------------------

515_Homarus_gammarus -------------------------------------------------

501_Mysis_diluviana -------------------------------------------------

520_Neomysis_americana -------------------------------------------------

530_Vanessa_cardui RAALYARFPALACQPSPEDNASVASAATAT-E---EKPSA---------

Homo_sapiens_GPR52 RLGLRRLSETMCTSCM-CVKDQEAQEPKPRKR---ANSCSI--------

Homo_sapiens_MTNR1A RKEYRRIIVSLCTARVF--FVDSSNDVADRVK---WKPSPLMTNNNVV-

Anolis_carolinensis_pinealOpsi RSCLLSTL---SCGRR-PQAAQGTTPAAISSP---RGRTLEGSRNKVVP

478_D_melanogasterRh1 RLALKEKCPCCVFGKVDDGKSSDAQSQATASE---AESKA---------

375_D_melanogasterRh4 RLELQKRCPWLGVNEKSGEISSAQSTTTQEQQ----QTTAA--------

Bostaurus_rhodopsin RNCMVTTL---CCGKN-PLGDDEASTTVSKTE---TSQVAPA-------
